# Integrated single-cell and bulk RNA sequencing analyses reveal a prognostic signature of cancer-associated fibroblasts in head and neck squamous cell carcinoma

**DOI:** 10.3389/fgene.2022.1028469

**Published:** 2022-12-08

**Authors:** Yichen Yang, Ben Ma, Litao Han, Weibo Xu, Xiaoxue Du, Wenjun Wei, Tian Liao, Qinghai Ji, Ning Qu, Yu Wang

**Affiliations:** ^1^ Department of Head and Neck Surgery, Fudan University Shanghai Cancer Center, Shanghai, China; ^2^ Department of Oncology, Shanghai Medical College, Fudan University, Shanghai, China; ^3^ Department of Otorhinolaryngology-Head and Neck Surgery, Zhongshan Hospital, Fudan University, Shanghai, China

**Keywords:** head and neck carcinoma, cancer-associated fibroblast, single-cell sequencing, prognostic signature, bulk RNA sequencing

## Abstract

**Objectives:** To identify a prognosis-related subtype of cancer-associated fibroblasts (CAFs) in head and neck squamous cell carcinoma (HNSCC) and comprehend its contributions to molecular characteristics, immune characteristics, and their potential benefits in immunotherapy and chemotherapy for HNSCC.

**Materials and Methods:** We performed single-cell RNA sequencing (scRNA-seq) analysis of CAFs from the samples of HNSCC patients derived from Gene Expression Omnibus (GEO), to identify the prognosis-related subtype of CAFs. CAFs were clustered into five subtypes, and a prognosis-related subtype was identified. Univariate and multivariate cox regression analyses were performed on the cohort selected from The Cancer Genome Atlas (TCGA) to determine signature construction, which was validated in GSE65858 and GSE42743. A prognostic signature based on 4 genes was constructed, which were derived from prognosis-related CAFs. The molecular characteristics, immune characteristics as well as the predicted chemosensitivity and immunotherapeutic response in the signature-defined subgroups were analyzed subsequently.

**Results:** The patients with higher CAF scores correlated with poor survival outcomes. Additionally, a high CAF score correlated with lower infiltration levels of many immune cells including M1 macrophages, CD8^+^ T cells, follicular T helper cells, monocytes, and naïve B cells. High CAF score also demonstrated different enrichment pathways, mutation genes and copy number variated genes. Furthermore, patients with high CAF scores showed lower sensitivity for chemotherapy and immunotherapy than those with low CAF scores.

**Conclusion:** The results of our study indicate the potential of the CAF signature as a biomarker for the prognosis of HNSCC patients. Furthermore, the signature could be a prospective therapeutic target in HNSCC.

## Introduction

Head and neck cancers comprise an array of cancers originating from the upper aerodigestive tract, with an incidence rate of sixth in the world (Leemans et al., 2011). Head and neck squamous cell carcinoma (HNSCC) is the most common histological type, with a 5-year survival rate of less than 50%. The traditional multimodality therapeutic strategy for HNSCC is the synthetic treatment of surgery, radiotherapy, and chemotherapy; however, this approach is less efficient for recurrent and metastatic tumors. In 2016, nivolumab and pembrolizumab, which are anti-programmed cell death 1 (anti-PD-1) antibody, were approved by the US Food and Drug Administration (FDA) for HNSCC treatment. Nonetheless, they are only effective in a select number of patients (Ferris et al., 2016; Larkins et al., 2017; Cohen et al., 2019). The molecular mechanisms underlying the tumorigenesis, progression, and metastasis of HNSCC have been continuously analyzed to identify novel therapeutic targets, but its intricacy cumbers traditional anti-cancer therapies widely use (Mroz and Rocco, 2016; Nishino et al., 2017). Nowadays, HNSCC is no longer regarded as merely malignant tumor cells but as a form of cancer with a complicated tumor microenvironment (TME) (Wu and Dai, 2017). During the early phase of TME researches, normal cells in TME were perceived as ideal targets for relative genetically stable (Joyce, 2005). As research continues, TME is no longer regarded as merely a tumor inhibitor but a double-edged sword which interacted with malignant cancer cell (Quail and Joyce, 2013). Nowadays, HNSCC is no longer regarded as merely malignant tumor cells but as a form of cancer with a complicated tumor microenvironment (TME), through which cells in the matrix interact with malignant cells to mediate tumorigenesis and metastasis (Wu and Dai, 2017).

Cancer associated fibroblasts (CAFs) are an essential part of the TME. As the major component of tumor stromal cells, CAFs have several origins, such as spontaneous mutation from normal fibroblasts, tumor cell-induced mutations, and transdifferentiating from epithelial or mesenchymal cells (Sahai et al., 2020). Following activation, CAFs provide physical support and contribute to tumor development and progression *via* various processes (Hanahan and Weinberg, 2011; Castells et al., 2012; Costa et al., 2018; Bertero et al., 2019; Mhaidly and Mechta-Grigoriou, 2020). Past studies have suggested that CAF enrichment leads to inconsistent prognosis by influencing different factors in various cancers (Özdemir et al., 2014; Costa et al., 2018; Monteran and Erez, 2019; Wang et al., 2021; Wen et al., 2021). The contradictory outcomes could be due to the heterogeneity of cancers and the unknown markers of CAFs. In previous studies, non-specific mesenchymal cell markers, such as smooth muscle actin ( α - SMA), fibroblast activation protein (*FAP*), and podoplanin (*PDPN*), were commonly used to evaluate CAFs. The diverse phenotypes and functions of CAFs indicate heterogeneity (Richards et al., 2017; Li et al., 2018; Wen et al., 2019). In HNSCC, CAFs interact with cancer cells during the processes of tumor progression and invasion, metabolic reprogramming, angiogenesis, immunodepression, and tumor therapy (Bienkowska et al., 2021). With the development of single-cell technology, single-cell RNA sequencing can be used to analyze the heterogenous cell population at a single cell resolution at the transcriptome level; this strategy was employed to study the heterogenous cells in the present study (Ren et al., 2018).

In this study, we aimed to understand the CAF and its potential as a prognostic biomarker for HNSCC patients. In addition, we aimed to gain insight into the molecular and immune characteristics of CAFs and their potential impact on the immunotherapy and chemotherapy of HNSCC patients.

## Material and methods

### Data sources and preprocessing

The scRNA-seq files were downloaded from GSE103322 (Puram et al., 2017), GSE164690 (Kürten et al., 2021) and GSE139324 (Cillo et al., 2020) *via* the Gene Expression Omnibus database (https://www.ncbi.nlm.nih.gov/geo/). The raw data were processed by standard way of Seurat (version 3.2.3). For each sample, the cells with less than 200 or more than 5,000 features were filtrated and the mitochondrial RNA percentage >5 were excluded.

The bulk of the RNA sequencing data were obtained from The Cancer Genome Atlas (TCGA) database with normalized reading counts (N = 502). In addition, RNA sequencing data were obtained from the GEO database in GSE65858 (N = 270) and GSE42743 (N = 74), which were used for external validation.

### Assessment of CAF infiltration level

CAF infiltration levels were evaluated with three algorithms, MCPCOUNTER (Becht et al., 2016), XCELL (Aran et al., 2017), and EPIC (Racle et al., 2017), using the TIMER2.0 online-tool (http://timer.cistrome.org/).

### CAF scRNA-seq analysis

We utilized the Seurat R package (version 3.2.3) to analyze CAFs separately (Butler et al., 2018). Using the resolutions 0.05, the clusters were determined with the FindClusters function. The principal component analysis was performed and visualized using the uniform manifold approximation and projection (UMAP) and t-Distributed Stochastic Neighbor Embedding (t-SNE) methods for dimension reduction. The scRNA-seq data were used to define five different subtypes of CAFs and their markers genes. The marker genes of each cluster were identified using the FindMarkers function, which was used to perform differential gene expression analysis between the cluster and all other cells using a Wilcoxon Rank-Sum test. All genes with false discovery rates (FDR) < 0.05 and |log Fold change|>1.0 (log FC) were regarded as marker genes. The top20 marker genes with largest logFC were list in Supplementary Table S1.

### Construction and validation CAF gene signatures

Top20 marker genes of each cluster were subjected to univariate cox regression analysis to select prognosis-related markers. The fraction of each cluster was calculated by geometric mean expression of prognosis-related genesand regarded as continuous variables. The results were then analyzed *via* multivariable regression with other clinicopathological factors to select the independent risk factors for prognosis. The CAF signatures were assessed by the summation of the expression of each gene multiplied by the coefficient of each gene. The median of signatures was considered as the cut-off value, and the prognostic power of the CAF signature was calculated using the log-rank tests; the results were visualized using Kaplan-Meier (K-M) survival curves for each cohort from TCGA and GEO.

### Gene set enrichment analysis in the Cancer Genome Atlas HNSC cohort

Patients were divided into two groups based on the median of CAF score. We explored the different hallmark gene sets between the high-CAF risk group and low-CAF risk group. Enrichment analysis was performed using the GSEA method based on HALLMARK gene sets with clusterProfiler package of R. The |normal enrichment score| (|NES|) > 1, nominal p value (NOM p-val) < 0.01, FDR q-val < 0.01 were regarded as significantly enriched pathways. The gene sets “h.all. v7.4. entrez” were downloaded from the Molecular Signatures Database (MSigDB). Single sample GSEA (ssGSEA) analysis was then performed on several enriched pathways using the GSVA package.

### Estimation of tumor microenvironment infiltration

Twenty-two types of immune cells were estimated using the CIBERSORTX (https://cibersortx.stanford.edu/) by using the signature genes from LM22. Differences between the two subgroups were compared using Wilcoxon test.

### Evaluation significant somatic mutations and copy number variations

The information about somatic mutation and CNV in the TCGA-HNSC cohort was downloaded from TCGA and cBioPortal (https://portal.gdc.cancer.gov and https://www.cbioportal.org/). The ‘Maftools’ package was used to summary mutations and perform the oncoplot to visualize the somatic mutations. For CNV analysis, the segment mean value of each region was used to determine amplification and deletion using 0.2 and -0.2. Chi-square test was used to compared CNV differences between the two groups, and the “ComplexHeatmap” package was used to visualized the results.

### Chemotherapeutic sensitivity and immunotherapeutic response prediction

The pRRophetic R package was used to predict the chemosensitivity of each patient. The half-maximal inhibitory concentration (IC50) in each patient was calculated by building a ridge regression model with ten-fold cross-validation (Geeleher et al., 2014).

The predicted immunotherapeutic effects were estimated by Tumor Immune Dysfunction and Exclusion (TIDE, http://tide.dfci.harvard.edu) (Jiang et al., 2018).

### Statistical analysis

All statistical analyses and visualization were performed using R software v3.6.3 (https://www.r-project.org/). *p* < 0.05 was regarded as statistical significance.

## Results

### CAF infiltrations in head and neck squamous cell carcinoma patients

**FIGURE 1:**
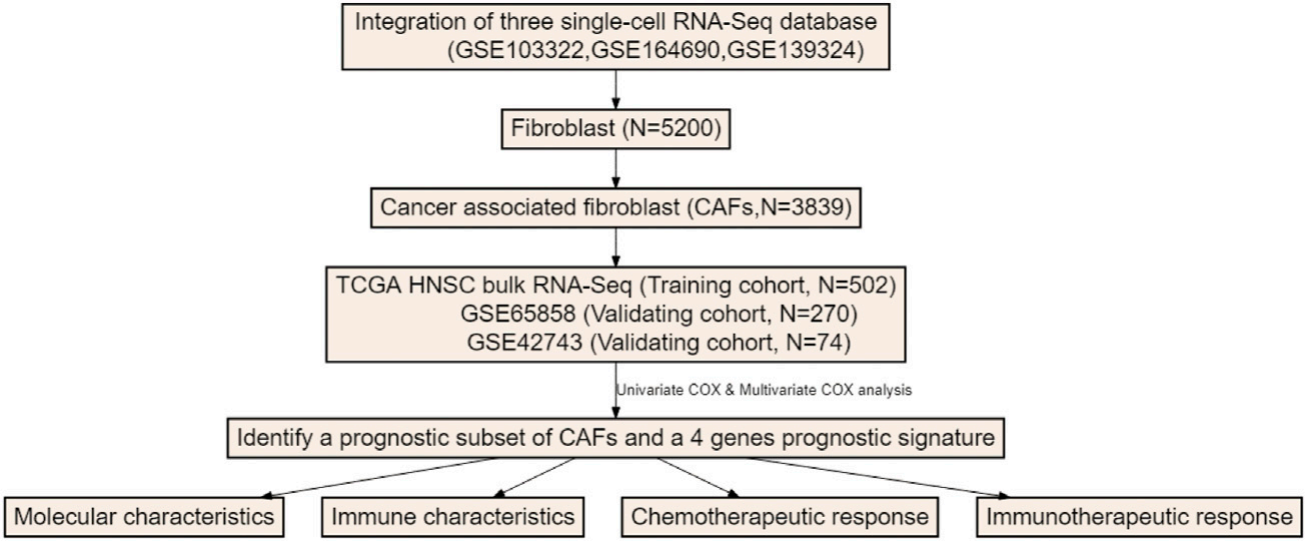
The flow chart of this study.

The overall study schematic flow chart is shown in Figure 1. To detect CAF abundances of each patient, three methods, MCP-COUNTER, XCELL and EPIC, were used to estimate the CAF score of each patient with HNSCC from the TCGA database. These three methods are all based on the transcriptome data but with distinctive algorithms. MCP-COUNTER is based on geometric mean of the expression of marker genes, XCELL is based on enrichment scores of single sample GSEA (ssGSEA) and EPIC is based on constrained least square regression. The estimated CAF scores were regarded as phenotype data for univariate cox regression analysis, which was performed to detect the relevancy of CAF scores as a prognostic tool for HNSCC patients and shown in Supplementary Figure S1. It shown that CAF scores calculated by these three algorithms cannot discriminate the patients with poor survival.

### Identification of different subtypes of cancer associated fibroblasts and its corresponding biomarkers

**FIGURE 2:**
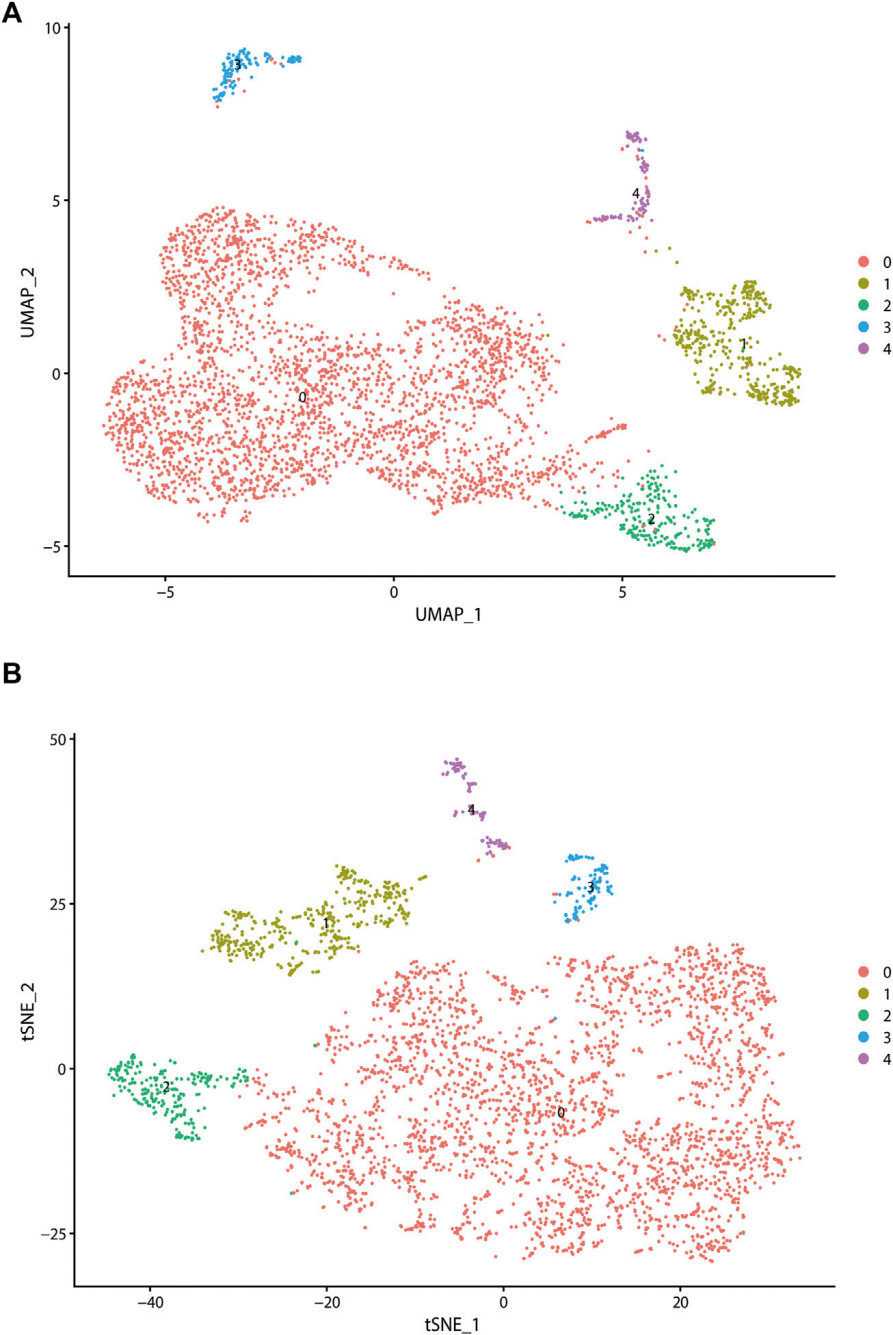
Analysis of single-cell RNA sequencing data in GSE103322, which including 18 primary HNSCC patients. All fibroblasts were isolated and clustered into 4 subtypes *via* t-SNE dimensionality reduction **(A)** and UMAP **(B)** dimensionality reduction.

To further explore the association between CAF infiltration and prognosis, we used the scRNA-seq data containing CAF information from GSE103322, GSE164690, and GSE139324. After quality control and normalization, all fibroblasts were annotated and isolated (N = 5,200). CAFs were annotated and perform sub-clustering analysis (N = 3,839) subsequently. The distributions of CAFs from GSE103322, GSE164690, and GSE139324 were shown in Supplementary Figure S2. It was divided into five subtypes finally. Two dimension-reduction methods (t-SNE and UMAP) were adopted to visualize the distribution of each subtype of CAFs. (Figures 2A,B). The top20 highly expressed genes (FDR < 0.05) across each subtype were regarded as markers and shown in Supplementary Table S1.

### Constructing CAF-related prognostic signature

**FIGURE 3:**
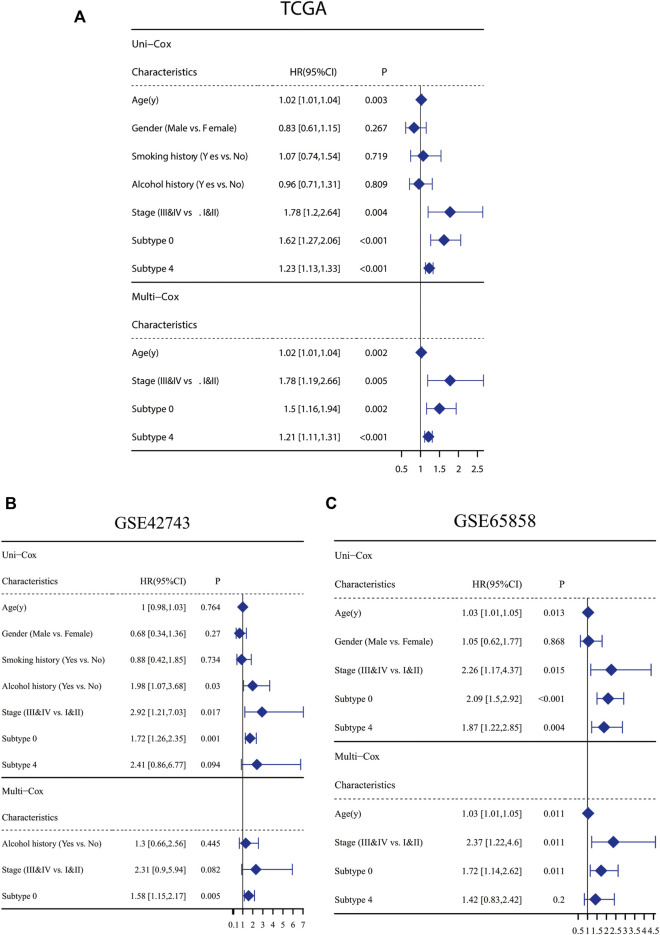
Clinicopathological and CAF infiltrations correlation of prognosis in HNSC patients. Univariate Cox analysis of clinicopathologic factors and each CAF subtype and multivariate Cox analysis of the significant factors (*p* < 0.05) in TCGA-HNSC cohort **(A)**, GSE65858 **(B)** and GSE42743 **(C)**.

**FIGURE 4:**
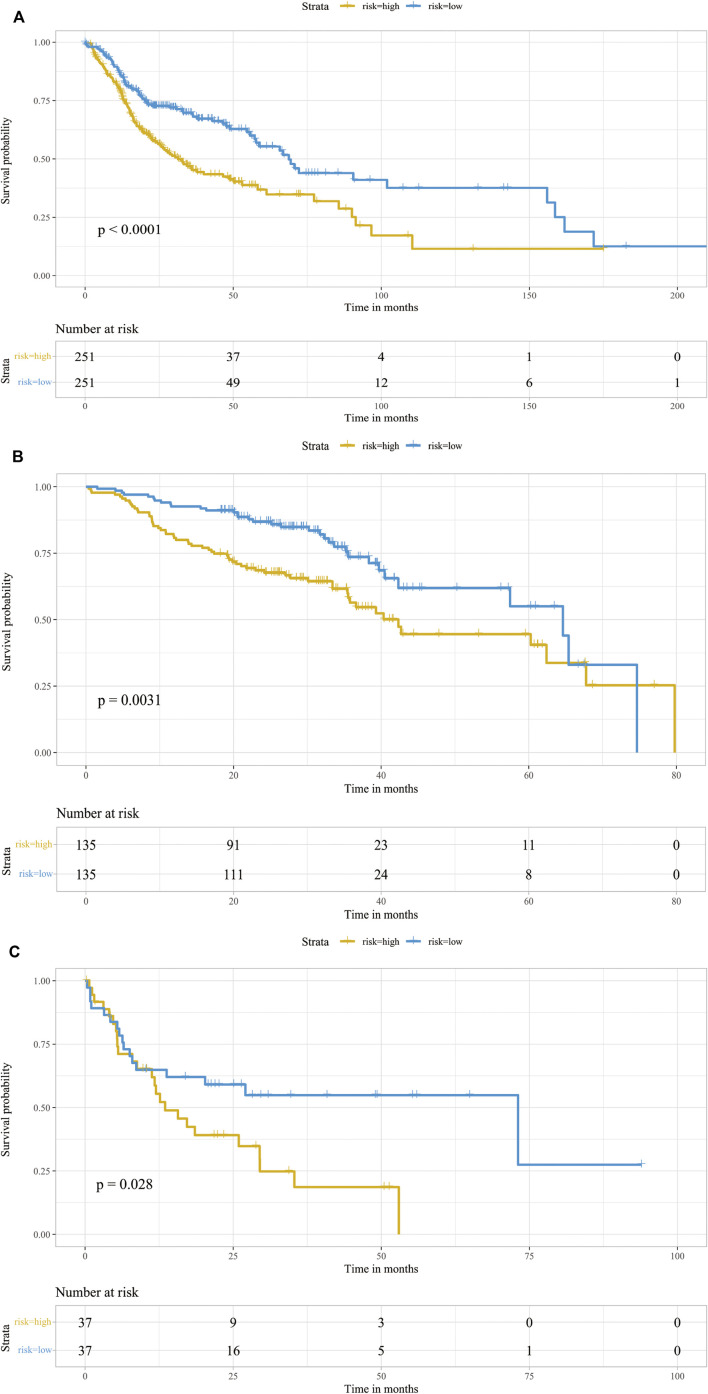
Prognostic analysis of the infiltrations of CAFs. Kaplan-Meier survival curve of CAF subgroups in TCGA-HNSC cohort **(A)**, GSE65858 **(B)** and GSE42743 **(C)**. Patients were stratified into two groups according to its CAF risk score. The table at the bottom showed the number of patients at risk in each group.

Markers of each subtype were included in the univariable cox regression analysis for the TCGA training cohort to detect prognosis-related markers (*p* < 0.01). Then, the fraction of each subtype was calculated using the selected prognosis-related markers. In TCGA database, only cluster0 and cluster4 have significant prognosis-related markers expression. To detect whether these CAF subtype can be regarded as an independent factor influencing prognosis, multivariate cox regression analysis was performed. For TCGA database, multivariate cox regression analyses showed that age, tumor stage, cluster0 and cluster4 of CAFs were significantly associated with prognosis (Figure 3A). The same analyses were performed for validating the GEO cohorts (GSE65858 and GSE42743), the results are shown in Figures 3B,C. In all datasets, CAF subtype 0 uniquely acted as an independent factor for poor prognosis. Therefore, we constructed a signature consisting of 4 marker genes from CAF subtype 0. The 4 genes included in our signature were Ferritin Heavy Chain 1 (*FTH1*), Transmembrane 4 L Six Family Member 1 (*TM4SF1*), Solute Carrier Family 16 Member 3 (*SLC16A3*) and Immediate Early Response 3 (*IER3*). The median risk score was used as the cut-off value to divide patients into two subgroups, low-CAF group and high-CAF group. The high-CAF group consistently correlated with low overall survival, which was evident for all cohorts compared with low-CAF group (Figures 4A–C, *p* < 0.05).

### Molecular characteristics of different CAF risk groups

**FIGURE 5:**
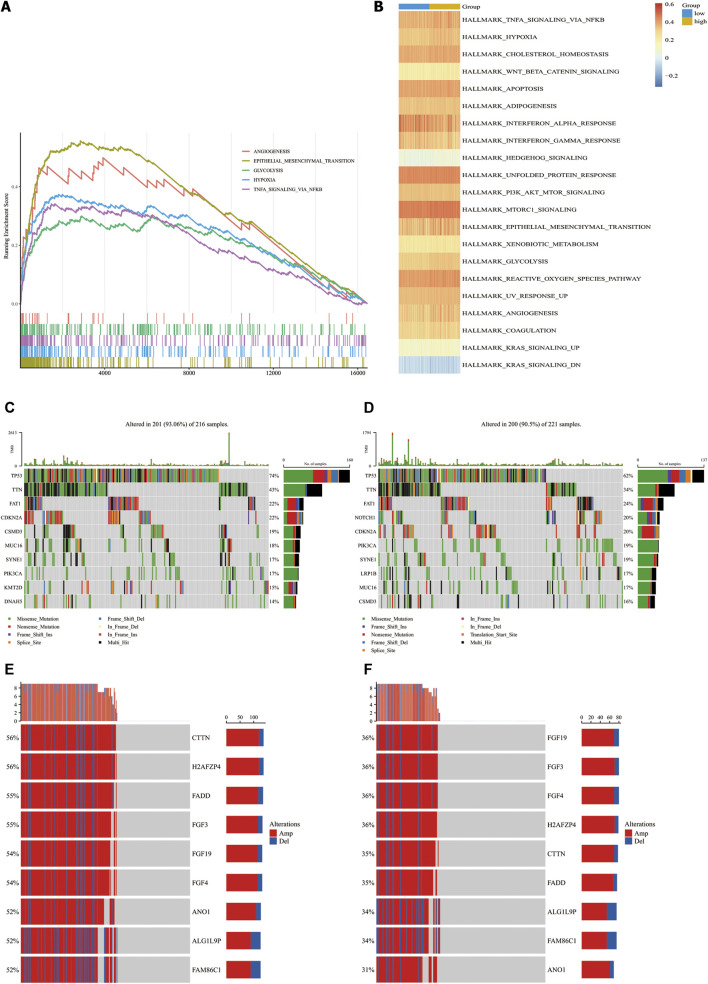
Molecular characteristics in CAF-high group and CAF-low group in TCGA-HNSC cohorts. **(A)**, Top4 tumor-associated gene sets enriched in CAF-high subgroup (FDR q-val<0.01). **(B)**, Heatmap of gene sets correlated significantly with CAF signature (*p* < 0.05). **(C,D)**, Waterfall plot illustrated the Top10 mutated genes in the TCGA-HNSC cohort patients in CAF-high groups **(C)** and CAF-low group **(D)**. Samples (central columns) are arranged to emphasize mutual exclusivity among mutations. The upper plot shows the mutation frequency of each patient. The right plot shows mutation percentage, the color coding in the bottom indicates the mutation types. **(E,F)**, Detailed information of top 10 genes with different copy number variation in CAF-high subgroups **(E)** and CAF-low group **(F)** (p-value of all genes are less than 0.0001). Samples (columns) in the central are arranged to emphasize copy number variated among patients. Each column in the upper represents a patient, the left percentages show the proportion of patients with mutated gene and the barplots in the right indicate the alteration types.

We performed GSEA using RNA-sequencing data from TCGA database to identify tumor-associated gene sets enriched in different CAF risk groups. As shown in Figure 5A, the top five tumorigenic pathways enriched in the high-CAF group were the epithelial mesenchymal transition pathway, angiogenesis pathway, hypoxia pathway, glycosis pathway and TNFα -NFκB signaling pathway (|NES| > 1, NOM p-val < 0.01, FDR q-val < 0.01). Next, we performed ssGSEA and determined the correlation between our CAF signatures and different pathways (Figure 5B). 21 pathways were found significantly related to our CAF signature scores (*p* < 0.01). Besides what mention above, many pathways related to the development of tumor enriched in CAF high group, including adipogenesis pathway, cholesterol homeostasis pathway, apoptosis pathway, PI3K-AKT pathway, mtorc1 signaling pathway. Meanwhile, interferon alpha response pathway and interferon gamma response pathway were enriched in CAF low group.

Next, we analyzed the associations of genetic mutations with CAF infiltration in TCGA-HNSC cohort. Patients were divided into two subgroups according to our CAF signature. In addition to nonsense mutations, missense mutations were the most common mutation type, followed by frameshift deletions. The top 10 genes with the highest mutation rates in each group are shown in Figures 5C,D. All the genes showed more than 10% mutation rates. *TP53*, *TTN* and *FAT1* mutated most frequently in both the groups. *TP53*, *TTN, FAT1, CDKN2A*, *CSMD3*, and MUC16 mutations were more common in high- CAF group while *SYNE1*, *PIK3CA*, *KMT2D*, and *DNAH5* are more common in low-CAF group. We also identified 203 significantly different mutation genes between two groups which shown in Supplementary Table S2. All of 203 genes mutated more frequently in high CAF group. It may suggest that high CAF infiltration related to a relative unstable state with more mutated genes.

Aberrant DNA CNVs are an important molecular mechanism during the occurrence and development of tumors. CNV information of the TCGA-HNSC cohort was obtained and compared using Chi-square test. The TCGA-HNSC cohort was divided into two subgroups according to pervious method. After deleting the very short CNV records,2,373 genes have significantly different CNV between two groups (adj.*p* < 0.05). The top 9 genes with the biggest disparity are shown in Figures 5E,F.

### Immune characteristics of high-CAF group and low-CAF group

**FIGURE 6:**
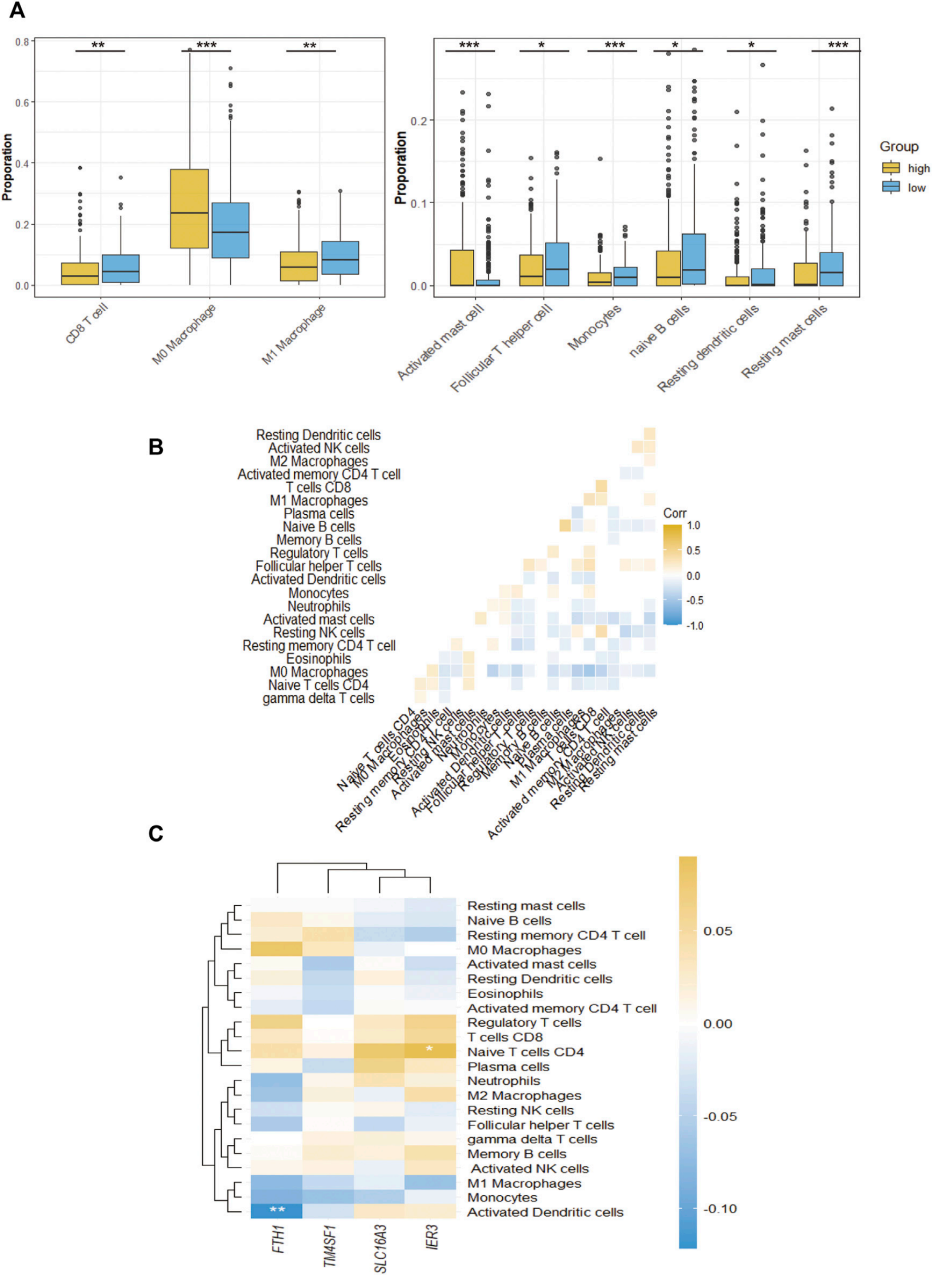
The TME immune cell infiltration characterization. **(A)**, The proportions of differently infiltrated TME immune cells in two CAF subgroups in TCGA-HNSC cohort. **(B)**, Correlations between CAF signature genes and different infiltrated TME immune cells. **(C)**, The correlation of all 24 infiltrated TME immune cells. *p-value < 0.05, **p-value < 0.01, ***p-value < 0.001, ****p-value < 0.0001.

To further distinguish which TME components result in distinct clinical outcomes, the constituents were compared between the two different CAF subgroups. Wilcoxon analysis was performed on two CAF subgroups of the TCGA-HNSC cohort to evaluate the differences in TME contents. Between the two groups, 9 types of TME cells showed significant differences. M0 macrophages, activated mast cells and eosinophils were significantly increased in the high-CAF group. In comparison, CD8 T cells, M1 macrophages, T follicular helper cells, naïve B cells, monocytes, resting dendritic cells and resting mast cells significantly increased in the low group (*p* < 0.05). The infiltration levels of these cells are shown in Figure 6A. The interactions between each type of immune cell are shown in Figure 6B. Our findings are consistent with those of previous studies, wherein patients with low CAF infiltration had a greater number of activated M1 macrophages and activated CD8+T cells which often exert anti-tumor effect in TME. This suggests that high CAF infiltrated in patients may related to immunosuppressive microenvironments. To further clarify the intrinsic factors influencing immune characteristics, the relationship between different infiltrated TME cells and CAF signatures were assessed, as shown in Figure 6C. In our signature, FTH1 was related to activated dendritic cell and IER3 was related to naïve B cell.

### Chemosensitivity and immunotherapy response prediction

**FIGURE 7:**
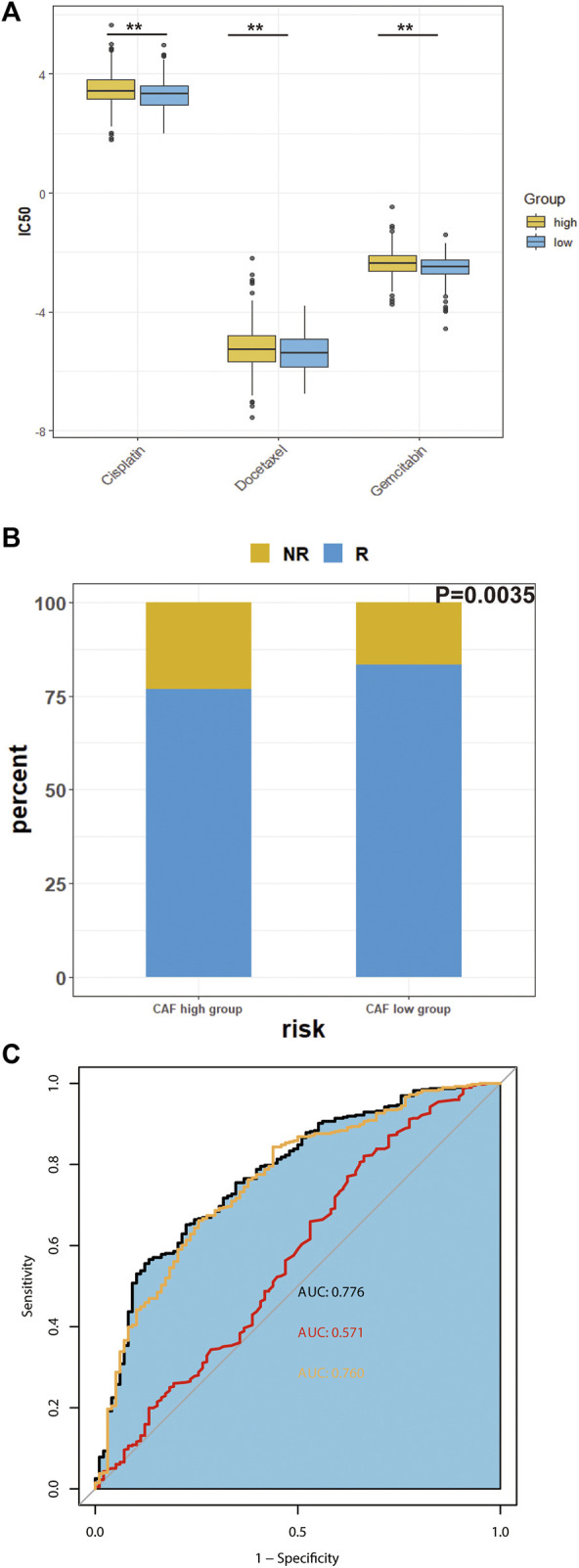
Predicted chemosensitivity and immunotherapeutic response in different CAF subgroups. **(A)**, The IC50 values of four anti-cancer drugs of different CAF-subgroup of TCGA-HNSC patients predicted by pPRophetic algorithm. **(B)**, Predicted immunotherapeutic effects of TCGA-HNSC **(C)**, The prediction effect of CAF signature. (Yellow represents the combination of many immune cell surface antigen including CD247, CD8 and Merck18; red represents TMB; black represents the combination of our CAF signature, TMB and cell surface antigens) *p-value < 0.05, **p-value < 0.01, ***p-value < 0.001, ****p-value < 0.0001.

We predicted the IC50 of three common chemotherapeutic agents used to treat HNSCC patients (cisplatin, docetaxel and gemcitabine) for the different CAF subgroups. IC50 is half maximal inhibitory concentration, which means the concentration that can inhibit half of total tumor cells. As shown in Figure 7A, the low-CAF group had a significantly lower IC50s for the chemotherapeutic agents (*p* < 0.05).

Next, we investigated the predicted immunotherapeutic effects of CAF signature in TCGA-HNSC cohort. As shown in Figure 7B, the percentage of patients who responded to immunotherapy was higher in the low-CAF group. The predictability of the efficacy of the CAF risk score in immunotherapy was visualized using the receiver operating characteristic curve (ROC) curve. According to previous studies, CAFs are involved in the resistance of immune checkpoints inhibitors (Bienkowska et al., 2021). As shown in Figure 7C, the integrated area under the curve (AUC) of the CAF signature, tumor mutation burden (TMB) and combined expression of CD8, CD274, and Merck18 was 0.776, which was higher than the AUC of TMB (AUC = 0.571) as well as the AUC of the expression of immune checkpoints (AUC = 0.760).

## Discussion

As a potential therapeutic target, CAF have been long studied. However, lack of specific biomarkers of CAFs has impeded their clinical application. In this study, we evaluated the total levels of CAF infiltration, which cannot be regarded as a risk factor of prognosis. We integrated three scRNA-seq databases including GSE103322, GSE164690, and GSE139324. Then, we evaluated the cancer associated fibroblast subtypes and their corresponding marker genes. We used the marker genes of each CAF subtype to identify the infiltration levels of CAFs in the TCGA-HNSCC cohort, GSE65858, and GSE42743. The univariate and multivariate cox regression analyses verified one subtype of CAF as an independent risk factor for overall survival, which we used to construct a CAF-related signature. Furthermore, we revealed the differences in immune and molecular characteristics between the two CAF subgroups.

The high-CAF group had low levels of anti-tumor immune cells, leading to a poor prognosis. These immune cells included CD8 T cells, M1 macrophages, T follicular helper cells, naïve B cells, monocytes, resting dendritic cells and resting mast cells. Some tumor-associated pathways, such as the angiogenesis, EMT, hypoxia, glycolysis and TNFα-NFκB pathways, were up-regulated in the CAF-high group. Moreover, the CAF-high group also had a significant higher rate of many gene mutations. Furthermore, the chemosensitivity and response to immunotherapy were predicted for the two CAF subgroups. Results showed that the CAF-high group was less sensitive to the conventional chemotherapeutic and immunotherapeutic agents compared with CAF-low group. Integration of the CAF signature and traditional indexes (TMB and immune cell surface antigens) can help in predicting the response to immunotherapy more accurately. This indicates that the CAF subtype signature could serve as not only a promising biomarker but also as a potential therapeutic target in HNSCC patients.

For the CAF-related 4 genes signature, none of them were identified as common CAF markers before. According to previous studies, *FTH1* is a subunit of ferritin, which regulate the ion metabolism and ferroptosis both *in vivo* and *in vitro* (Fang et al., 2021; Kong et al., 2021; Zhang et al., 2022). IER3 was proved mediated cancer development in many cancers including tongue cancer (Garcia et al., 2014; Xiao et al., 2019; Liu et al., 2021). *TM4SF1* was proved not only mediating the development of cancer but also the immunotherapeutic sensitivity. There are also many clinical trials of mAb-L6, which is the inhibitor of *TM4SF1*(Fu et al., 2020). *SLC16A3* was also proved could influence immunotherapeutic sensitivity by modulating lactate metabolism (Li et al., 2020; Fang et al., 2022).

As shown in the results, the overall infiltration level of CAF cannot be regarded as a risk factor for prognosis in HNSCC patient. In this study, we innovatively identified a subtype of CAF to improve the prognosis and therapeutic outcome of HNSCC. However, there are some limitations to this study. First, it is a retrospective study based on public databases. The samples obtained from GSE103322 are not sufficient. Secondly, we defined the CAF subtypes on mRNA level but not protein level, which could be investigated in future studies. Finally, the CAF signature in this study is composed of 17 genes. Future research could simplify the signature by detecting the underlying mechanisms of each gene. More critically, there is a challenge of moving RNA-seq to the clinic. The variation of the biopsy and subsequent processing may cause extensive variation in transcriptome. Recent years, some sequencing methods with simplifier pre-processing have emerged such as single nuclei RNA sequencing. The liquid biopsy has also emerged with extensive attention. Compared to classical tissue biopsy, liquid biopsy can get rid of selection bias caused by spatial limitations and tumor heterogeneity. The study of Duda et al. firstly revealed that the TME components in circulation together with circulating tumor cells (Duda et al., 2010). Subsequent a series of studies demonstrated the existence of circulating CAFs or CAF-derived exosomes in body fluids (Wintzell et al., 2012; Ao et al., 2015; Herrera et al., 2019). The exact biomarkers of circulating CAF to predict prognosis and immune therapeutic effects in HNSCC are deserving of further study.

## Data Availability

The datasets presented in this study can be found in online repositories. The names of the repository/repositories and accession number(s) can be found below: https://www.ncbi.nlm.nih.gov/geo/, GSE103322, GSE164690, GSE139324, GSE65858, and GSE42743; https://portal.gdc.cancer.gov/, TCGA-HNSC.

## Supplementary material

Supplementary Table S1 Top20 marker genes of each subtype of cancer associated fibroblasts.

Supplementary Table S2 Significantly different mutation genes between two groups.

Supplementary Figure S1 Univariate Cox analysis of whole level CAF infiltration. CAF infiltration was estimated by MCP-COUNTER, XCELL and EPIC.

Supplementary Figure S2 The distribution of merged CAFs from GSE103322, GSE139324 and GSE164690 via UMAP dimensionality reduction **(A)** and tSNE dimensionality reduction **(B)**.
