# Supplementary figures and images for: Integrated single-cell and bulk RNA sequencing analyses reveal a prognostic signature of cancer-associated fibroblasts in head and neck squamous cell carcinoma
Source: Front Genet. 2022 Dec 8;13:1028469. doi: 10.3389/fgene.2022.1028469 (PMC9775281; doi:10.3389/fgene.2022.1028469)

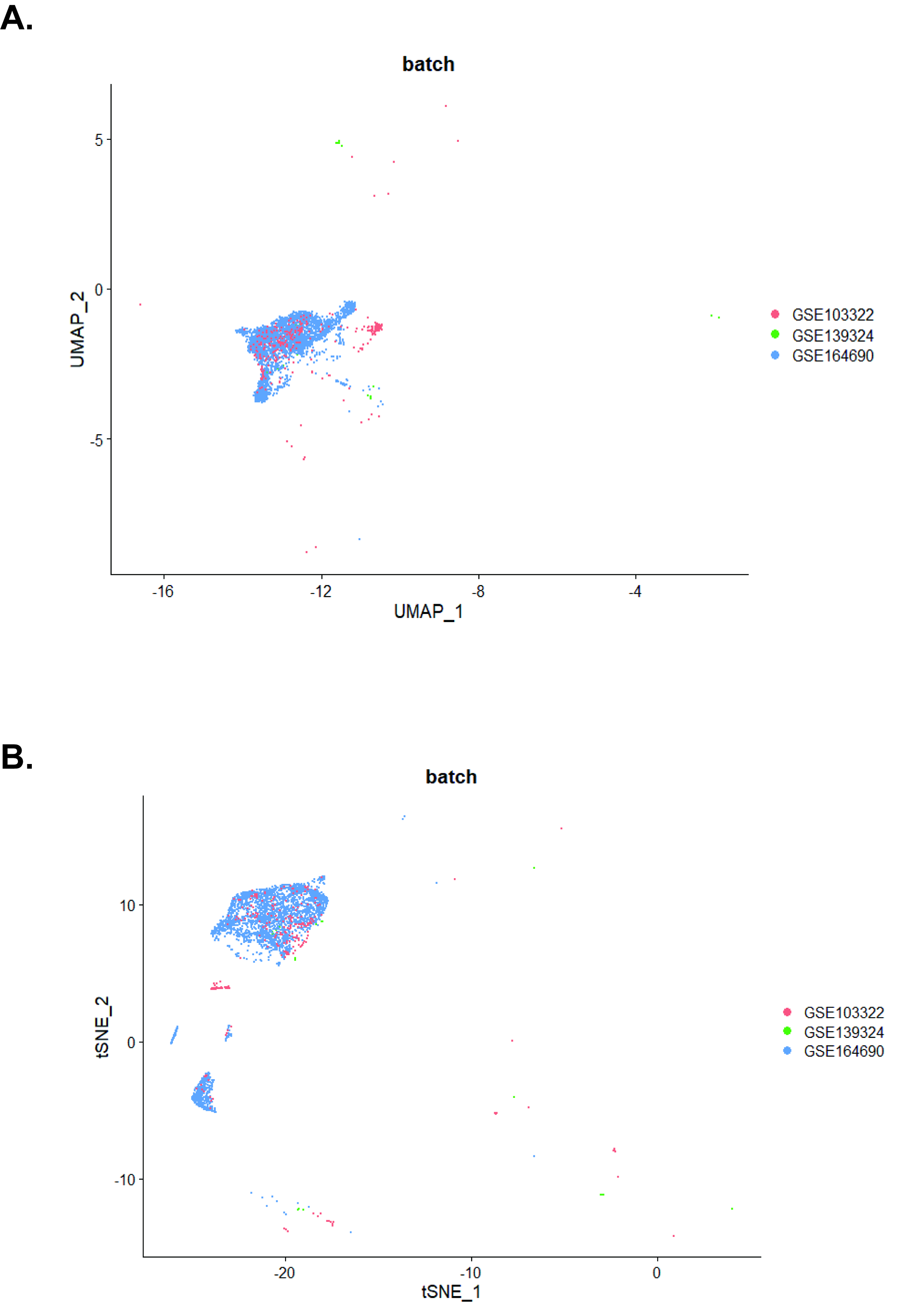

Supplement: Supplementary file 2 [file Image1.TIF]

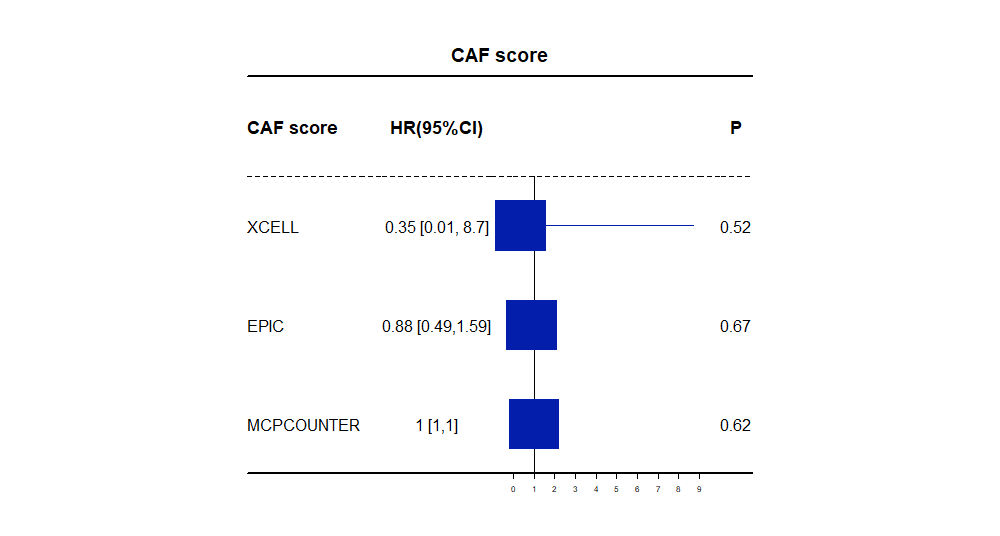

Supplement: Supplementary file 4 [file Image2.TIFF]
